# Meta-analysis of risk factors for recurrence in cervical cancer patients following fertility-sparing treatment

**DOI:** 10.3389/fsurg.2025.1625067

**Published:** 2026-02-05

**Authors:** Rui Shi, Weili Hou, Yanlei Gao, Suli Sun, Jia Ling, Yaru Ma

**Affiliations:** 1 Gynecology Department, Shijiazhuang Maternity & Child Healthcare Hospital, Shijiazhuang, Hebei Province, China; 2 Maternal and Child Service Center, Shijiazhuang Maternity & Child Healthcare Hospital, Shijiazhuang, Hebei Province, China; 3 Radiology Department, Shijiazhuang Maternity & Child Healthcare Hospital, Shijiazhuang, Hebei Province, China; 4 Science and Education Section, Shijiazhuang Maternity & Child Healthcare Hospital, Shijiazhuang, Hebei Province, China; 5 Family Planning Department, Shijiazhuang Maternity & Child Healthcare Hospital, Shijiazhuang, Hebei Province, China; 6 Gynecology Clinic, Shijiazhuang Maternity & Child Healthcare Hospital, Shijiazhuang, Hebei Province, China

**Keywords:** cervical cancer, fertility-sparing treatment, recurrence, risk factors, meta-analysis

## Abstract

**Background:**

To identify and evaluate the main risk factors for recurrence in cervical cancer patients who undergo fertility- sparing treatment.

**Methods:**

A comprehensive search of multiple databases, including PubMed, Embase, and Web of Science, was performed to identify studies assessing the recurrence risk in cervical cancer patients treated with fertility-sparing procedures. Data from eligible studies were pooled, and the relative risks (RR) with 95% confidence intervals (CI) were calculated to evaluate the association between various risk factors and recurrence risk.

**Results:**

Ten studies met inclusion criteria. Recurrence risk was significantly higher in patients aged ≤30 years (RR: 2.03, 95% CI: 1.89–2.19), those with tumor size ≥2 cm (RR: 1.94, 95% CI: 1.82–2.06), stage > IA within the fertility-sparing spectrum (RR: 2.46, 95% CI: 2.29–2.64), lymphovascular space invasion (LVSI) positivity (RR: 2.09, 95% CI: 1.90–2.30), and lymph-node metastasis (RR 3.10, 95% CI 2.76–3.48). Heterogeneity was low across comparisons $\left(I^{2}\approx 0\text{\%}\right)$, and no significant small-study effects were detected.

**Conclusion:**

Age ≤30 years, tumor size ≥2 cm, stage > IA, LVSI positivity, and lymph-node metastasis are robust predictors of recurrence following fertility-sparing treatment in cervical cancer. Incorporating these variables into preoperative counseling, operative strategy, and follow-up planning may enhance oncologic safety while preserving reproductive potential in appropriately selected patients. Our findings are consistent with current guideline recommendations, which generally limit fertility-sparing approaches to tumors ≤2 cm, while tumors exceeding this threshold require cautious consideration and are usually not regarded as appropriate candidates outside of clinical trials or exceptional multidisciplinary contexts.

## 1 Introduction

Cervical cancer is one of the most common malignancies affecting women globally, ranking as the fourth most prevalent cancer and the fourth leading cause of cancer-related deaths among women (1, 2). According to the World Health Organization (WHO), cervical cancer causes over 300,000 deaths annually, with the highest incidence found in low- and middle-income countries, where access to effective screening and vaccination programs is limited (3, 4). Despite advances in early detection methods, such as Pap smears and HPV testing, and the widespread introduction of HPV vaccines targeting high-risk HPV types, cervical cancer remains a major public health challenge, particularly among young women (5). The disease is most diagnosed in women of reproductive age, many of whom are concerned about preserving their fertility during cancer treatment.

Fertility-sparing treatments have become a crucial option for young women diagnosed with early-stage cervical cancer (6). These treatments aim to balance effective oncological management with the preservation of fertility. Common fertility-preserving procedures, such as conization, loop electrosurgical excision procedure (LEEP), and radical trachelectomy, have been shown to offer favorable oncological outcomes while maintaining reproductive function (7–9). However, fertility-sparing treatments are not without risks, and recurrence remains a significant concern (9). The risk of recurrence after fertility-sparing treatment is influenced by various factors, including tumor characteristics, surgical methods, and pathological features (10–12). These factors can significantly affect both the prognosis and fertility outcomes of patients.

Cervical cancer recurrence not only threatens survival but also compromises the possibility of future pregnancies. Recurrence often requires more invasive treatments, such as hysterectomy, radiation therapy, or chemotherapy, all of which result in the loss of fertility (13). Furthermore, the psychological burden of recurrence in young women can be profound, leading to emotional distress and long-term reductions in quality of life (14, 15). As more young women opt for fertility-preserving treatments, understanding the factors' influencing recurrence is critical for improving patient outcomes, optimizing follow-up care, and providing counseling on fertility preservation.

The primary objective of this study is to identify and assess the key risk factors associated with recurrence in cervical cancer patients who have undergone fertility-sparing treatments. By performing a meta-analysis of existing studies, we aim to synthesize data on clinical and pathological variables that may influence recurrence risk, including age, tumor size, tumor stage, lymph vascular space invasion (LVSI), and lymph node involvement. These factors have been inconsistently reported in the literature, and their role in predicting recurrence after fertility-sparing treatment remains unclear. This analysis will provide a clearer understanding of how these risk factors influence recurrence risk and offer valuable insights into clinical decision-making for young women with cervical cancer.

## 2 Methods

### 2.1 Study design and objective

A comprehensive literature search was conducted across multiple databases, including PubMed, Cochrane Library, Web of science and Embase. The search terms used included “cervical cancer,” “fertility-sparing treatment,” “recurrence,” and “risk factors,” combined using Boolean operators (AND, OR) to ensure an exhaustive retrieval of relevant studies. The search was limited to articles published in English and Chinese from January 2000 to December 2023, to ensure the inclusion of the most current and relevant research. Studies were considered eligible if they focused on patients with cervical cancer who underwent fertility-sparing treatment and reported outcomes related to recurrence and associated risk factors. No restrictions were placed on the study design, and both observational studies and randomized controlled trials (RCTs) were included in the analysis. The systematic review and meta-analysis protocol was prospectively registered in the PROSPERO database (International Prospective Register of Systematic Reviews), with registration number CRD42024599867.

### 2.2 Inclusion criteria

Studies were included in the analysis if they met the following criteria: (1) Clinical research designs, including randomized controlled trials (RCTs), cohort studies, and case-control studies, were considered eligible for inclusion. These study types were chosen to provide robust evidence regarding the association between fertility-sparing treatment and recurrence risk in cervical cancer patients. (2) The study population comprised women diagnosed with cervical cancer who underwent fertility-sparing treatments, such as conization, trachelectomy, or other fertility-preserving surgical interventions. (3) The study reported outcomes related to the recurrence of cervical cancer and identified potential risk factors associated with recurrence. This included both clinical and pathological factors, such as tumor size, lymph node involvement, margin status, and histological type. Studies that did not provide data on recurrence or lacked detailed information on associated risk factors were excluded from the analysis.

### 2.3 Exclusion criteria

Studies were excluded from the analysis if they met any of the following criteria: (1) Irrelevant studies that did not focus on the recurrence of cervical cancer or fertility-sparing treatment. This includes studies that primarily addressed other aspects of cervical cancer management, such as palliative care or non-fertility-preserving therapies. (2) Studies with incomplete or inadequate data, such as missing critical information on recurrence outcomes or risk factors. (3) Studies with poor methodological quality, as assessed by standard tools such as the Cochrane Risk of Bias tool for randomized trials or the Newcastle-Ottawa Scale for observational studies.

### 2.4 Data extraction

Data were independently extracted by two reviewers using a standardized data extraction form to ensure consistency and accuracy. The following key variables were extracted from each included study: (1) Study characteristics: author(s), publication year, and study design (e.g., randomized controlled trials, cohort studies, case-control studies). (2) Patient demographics and clinical features: sample size, age range, and any relevant baseline characteristics of the study population (e.g., disease stage, histological subtype, etc.). (3) Treatment details: specific fertility-sparing treatment(s) used, such as conization, trachelectomy, or other fertility-preserving procedures. (4) Follow-up data: follow-up duration and any reported outcomes related to recurrence. (5) Recurrence-related outcomes: recurrence rates, including time to recurrence and sites of recurrence, where available. Any missing or unclear data were clarified by contacting the corresponding author of the study when necessary.

### 2.5 Statistical analysis methods

Data analysis was performed using R software (version 4.3.1). This evaluation aimed to identify potential sources of bias in study design, patient selection, outcome reporting, and other methodological aspects that could influence the validity of the results. Effect sizes were quantified by calculating relative risks (RRs) and their corresponding 95% confidence intervals (CIs) to assess the association between fertility-sparing treatments and cervical cancer recurrence. The heterogeneity of the studies was assessed using the *I*^2^ statistic, which quantifies the percentage of total variation across studies that is due to heterogeneity rather than chance. An *I*^2^ value of 0%–40% was considered low, 30%–60% moderate, and 50%–100% high heterogeneity. In the presence of significant heterogeneity, a random-effects model was used to calculate pooled effect estimates. Sensitivity analysis was conducted to test the robustness of the results by excluding studies with high risk of bias or small sample sizes. If the overall findings remained consistent after the exclusion of certain studies, this would indicate the robustness and reliability of the results. All statistical tests were two-sided, with a significance level set at *p* < 0.05.

## 3 Results

### 3.1 Study selection and inclusion

**Figure 1:**
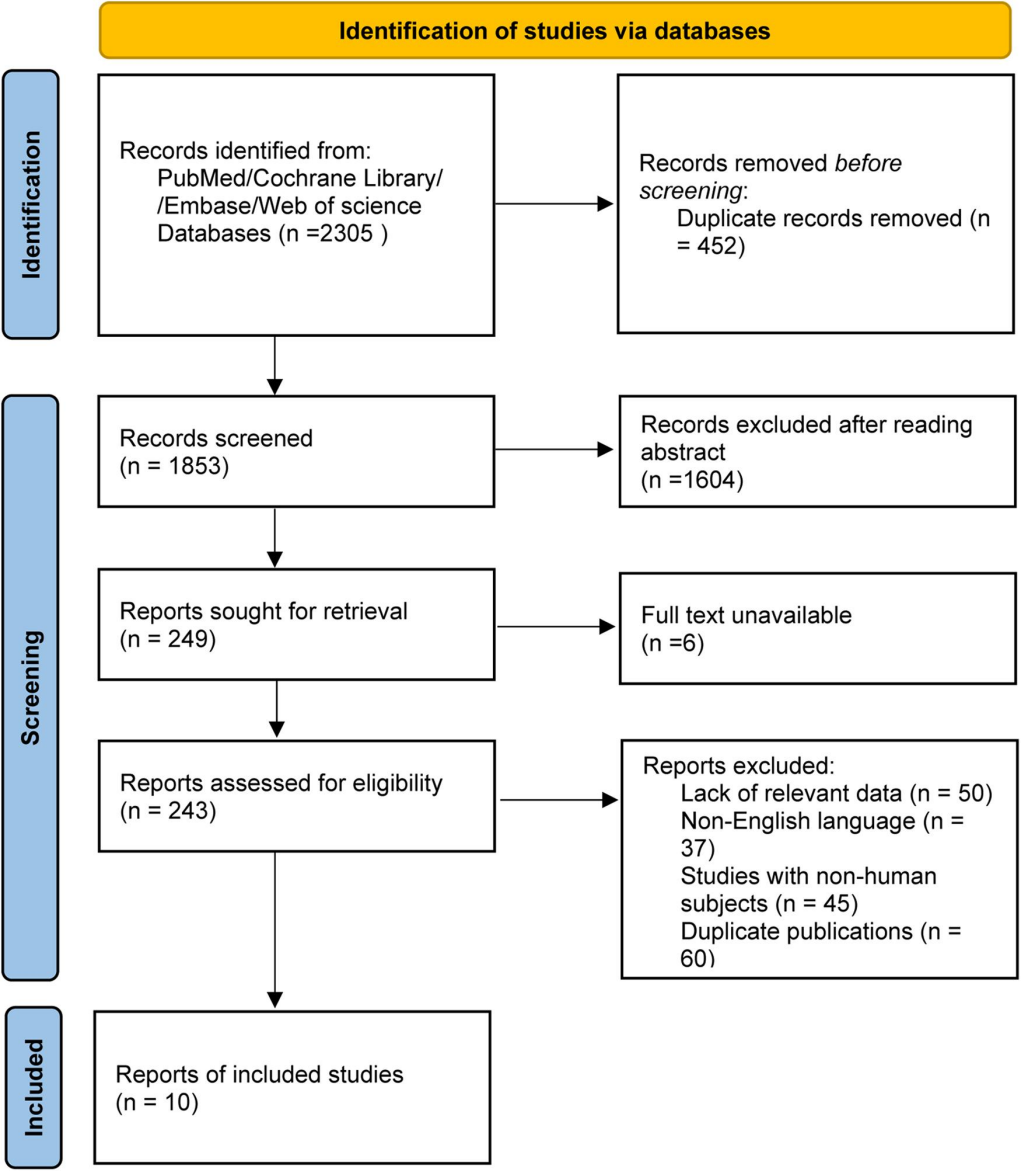
PRISMA flow diagram of literature screening.

The literature selection process in this study strictly adhered to the PRISMA (Preferred Reporting Items for Systematic Reviews and Meta-Analyses) guidelines, and a PRISMA literature screening flow diagram was constructed to visually present the entire selection process (Figure 1). The detailed steps of literature screening are as follows: A comprehensive search was conducted across multiple databases (including PubMed, Cochrane Library, Web of Science, and Embase), initially yielding a total of 2,305 potentially eligible studies related to the research topic. First, 415 duplicate studies were excluded through database deduplication tools and manual verification, leaving 1,890 studies for further screening.

In the first round of screening, the titles and abstracts of the remaining 1,890 studies were reviewed. Studies that were irrelevant to the research theme (e.g., focusing on non-fertility-sparing treatments for cervical cancer or non-recurrence outcomes), published in languages other than English or Chinese, or with unclear research objectives were excluded, resulting in 243 studies being retained for full-text assessment.

During the full-text evaluation phase, studies were excluded for the following specific reasons: (1) lack of key outcome measures related to cervical cancer recurrence (*n* = 26), such as studies that only reported fertility outcomes without mentioning recurrence; (2) inability to extract valid data on recurrence risk factors (*n* = 31), including studies with incomplete data tables or unreported effect sizes; (3) non-original research types (*n* = 9), such as reviews, meta-analyses, case reports, or commentaries that did not provide primary research data. After excluding 233 studies based on the above criteria, 10 studies that fully met the inclusion criteria were finally included in the meta-analysis.

Regarding other basic characteristics of the included studies, such as tumor size distribution, clinical staging of patients, and the incidence of lymph node metastasis among participants, detailed information is provided in Table 1 to facilitate readers' understanding of the baseline characteristics of the research population.

**Table 1:** Study characteristics and patient demographics.

Study (Author, Year)	Country	Study design	Sample size (n)	Follow-up (months)	Age ≤30 (%)	Age >30 (%)	Socioeconomic index (%)	Type of excisional treatment (%)	Lesion size (cm)	Surgical specimen depth (mm)	Concurrent CIN2/3 (%)	Follow-up co-testing (Cervical Cytology and HPV)	Hysterectomy performed (%)	Histology (%)	LVSI (%)	Nodal assessment (%)
Ekdahl et al. (10)	International	Retrospective study	166	64 (2–140)	NA	NA	NA	CKC (22.4%), LEEP (74.5%)	<1.5 cm (NA%), ≥1.5–2 cm (NA%)	NA	NA	NA	NA	SCC (59%), AC (41%)	NA	NA
Li et al. (11)	China	Retrospective study	333	32 (19–42)	45	55	Least disadvantaged (30.5%)	CKC (52.0%), LEEP (48.0%)	<1.5 cm (60.4%), ≥1.5–2.0 cm (39.6%)	Mean: 13.1 (1–40)	62.8% (present)	Yes (87.8%), No (12.2%)	Yes (16.9%)	SCC (52%), AC (48%)	60.40%	SLN (66%), PLND (34%)
							More disadvantaged (19.2%)									
Munro et al. (12)	Australia	Retrospective study	360	NA	45	55	Least disadvantaged (30.5%)	CKC (44%), LEEP (56%)	<1.5 cm (NA%), ≥1.5–2.0 cm (NA%)	NA	NA	NA	Yes (83.1%)	SCC (54%), AC (9%)	39.60%	NA
							Most disadvantaged (9.7%)									
Nica et al. (16)	Canada	Retrospective multi-centre cohort analysis	197	57 (31–69)	16%	84%	NA	VRT (39.2%), RRT (60.8%)	<2 cm (82.3%), >2 cm (12.7%)	NA	NA	NA	NA	SCC (59.5%), AC (40.5%)	63.30%	SLN (87%)
Park et al. (13)	Korea	Retrospective multicenter cohort study	88	NA	31 (20–40)	48 (60.8%)	NA	VRT (39.2%), RRT (60.8%)	<2 cm (82.3%), > 2 cm (12.7%)	NA	NA	NA	NA	SCC (59.5%), AC (40.5%)	63.30%	SLN (87%)
Schmeler et al. (17)	International	Prospective single-arm multicenter study	100	NA	33 (33%)	67 (67%)	NA	Laparoscopic (83%), Robotic (13%), Open (4%)	<2 cm (83%), > 2 cm (13%)	NA	50% (present)	Full lymph node dissection (58%), Sentinel lymph node biopsy + full lymph node dissection (38%)	Sentinel lymph node biopsy alone (4%)	SCC (48%), AC (52%)	NA	NA
Slama et al. (14)	International	retrospective observational multicenter study	733	NA	0.66	0.34	NA	CKC (52%), LEEP (48%)	<1.5 cm (60%), ≥1.5–2.0 cm (40%)	NA	NA	NA	SCC (59%), AC (41%)	NA	0.704	SLN (65.2%), PLND (34.8%)
Speiser et al. (15)	Germany	retrospective study	300	95 (31–234)	NA	NA	NA	CKC (NA%), LEEP (NA%)	<1.5 cm (NA%), ≥1.5–2.0 cm (NA%)	NA	NA	NA	SCC (NA%), AC (NA%)	NA	NA	NA
Yuan et al. (18)	China	Retrospective study	53	NA	0.375	0.625	NA	CKC (NA%), LEEP (NA%)	<2 cm (82%), ≥2 cm (18%)	NA	NA	NA	SCC (68%), AC (32%)	0.5	SLN (NA%)	
Zusterzeel et al. (19)	Netherlands	retrospective cohort study	132	NA	0.24	0.76	NA	CKC (NA%), LEEP (NA%)	<1.5 cm (NA%), ≥1.5–2.0 cm (NA%)	NA	50% (present)	Full lymph node dissection (70%)	SCC (NA%), AC (NA%)	NA	NA	

### 3.2 Meta-analysis results

#### 3.2.1 Association between age (≤30 years) and recurrence

**Figure 2:**
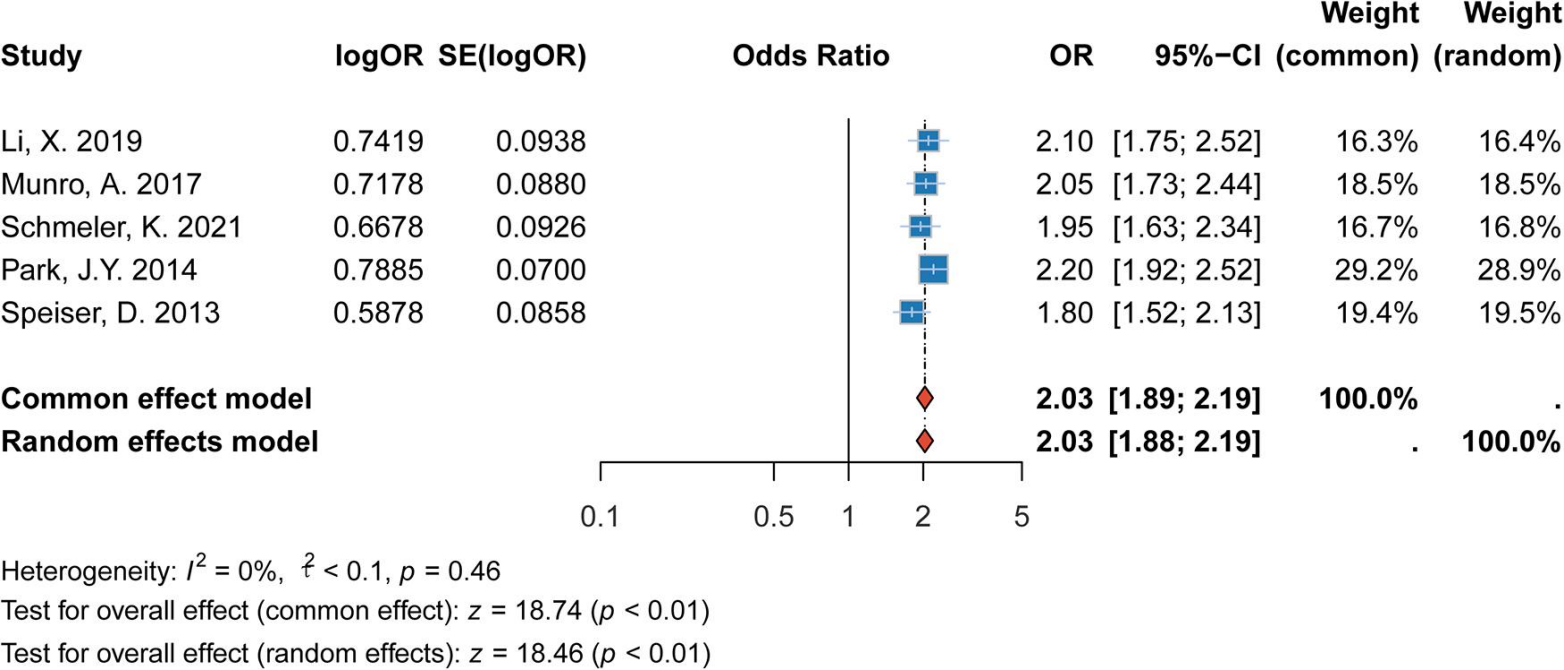
Forest plot of the association between age ≤30 years and recurrence risk.

The meta-analysis revealed that patients aged ≤30 years had a significantly higher risk of recurrence compared to those aged >30 years. The pooled relative risk (RR) was 2.03 (95% CI: 1.89–2.19, *p* < 0.001), indicating that younger patients had more than twice the risk of recurrence. This finding was consistent across multiple studies, and the heterogeneity between the studies was moderate (*I*^2^= 0%). The data suggest that age ≤30 years is an important factor to consider in predicting recurrence risk in cervical cancer patients (Figure 2).

#### 3.2.2 Association between tumor size (≥2 cm) and recurrence

**Figure 3:**
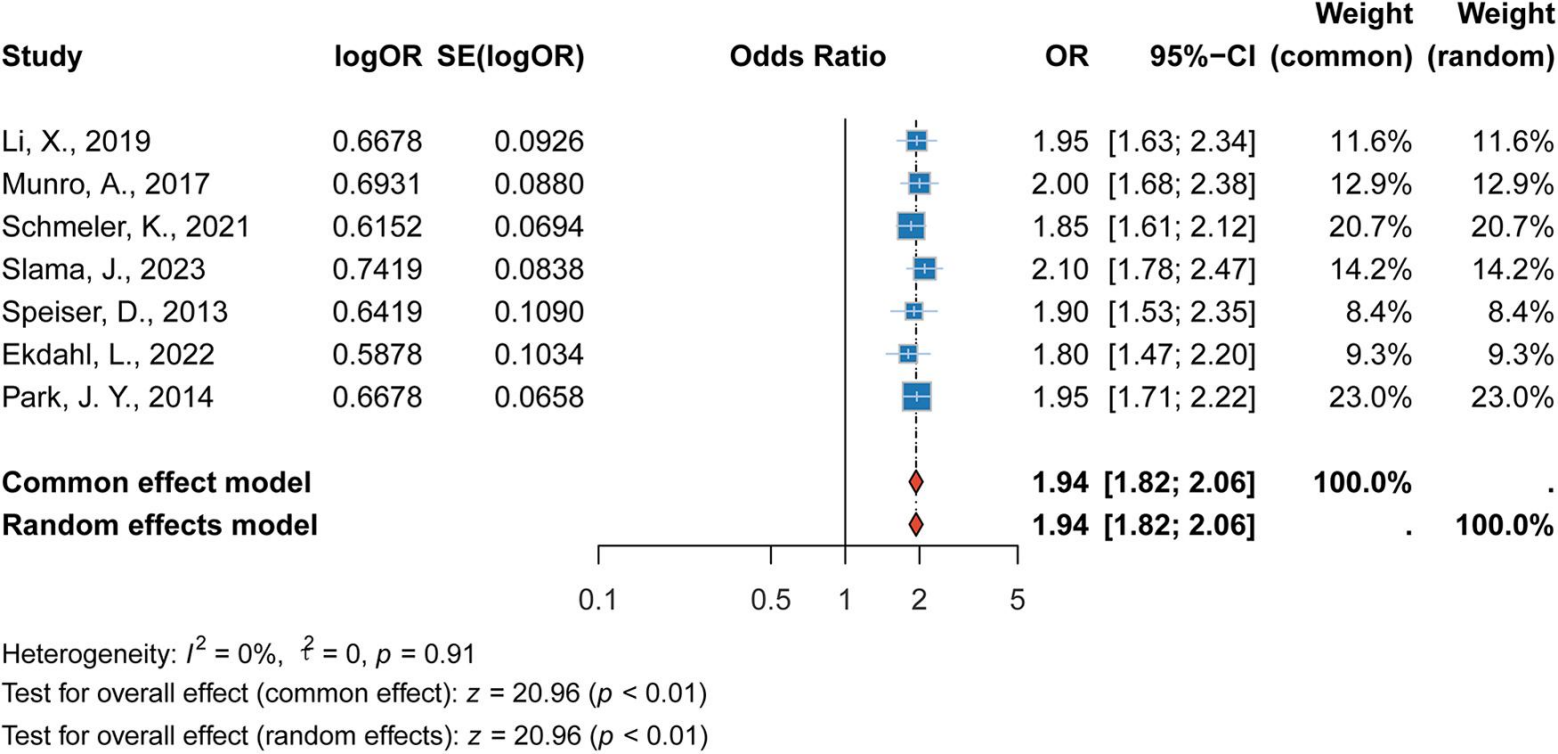
Forest plot of the association between tumor size ≥2 cm and recurrence risk.

The analysis showed that tumor size ≥2 cm was a significant predictor of recurrence. The pooled RR was 1.94 (95% CI: 1.82–2.06, *p* < 0.001), indicating that patients with larger tumors were at a significantly higher risk of recurrence. This finding was robust, with low heterogeneity observed among the included studies (*I*^2^ = 0%).While this finding corroborates existing evidence that tumor size is a critical prognostic factor in cervical cancer, it also aligns with current guideline recommendations that generally restrict fertility-sparing approaches to tumors ≤2 cm. For tumors exceeding this threshold, fertility preservation should be considered with great caution and typically only within clinical trial settings or after thorough multidisciplinary evaluation (Figure 3).

#### 3.2.3 Association between advanced stage (>IA) and recurrence

**Figure 4:**
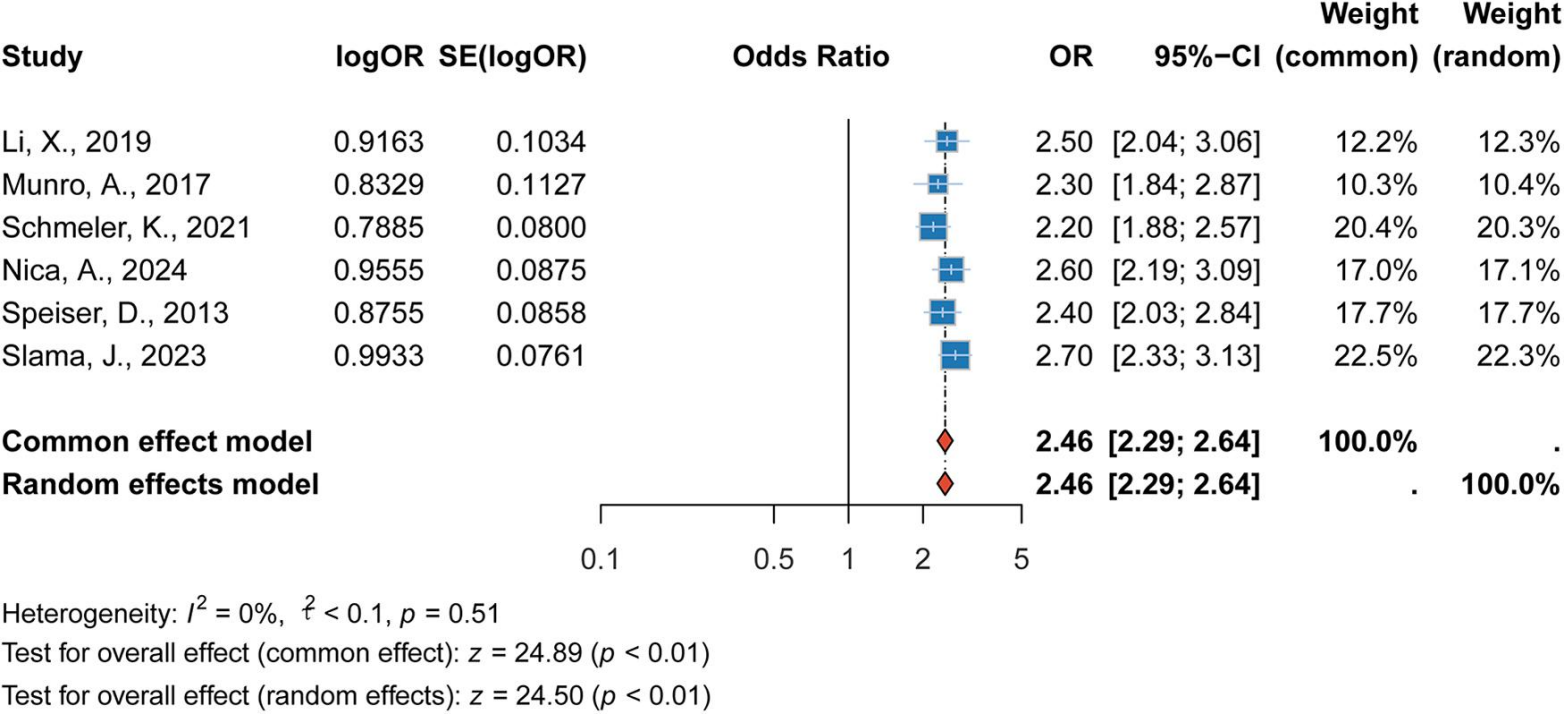
Forest plot of the association between advanced stage (>IA) and recurrence risk.

Our meta-analysis demonstrated that patients with advanced-stage cervical cancer (stage > IA) had a significantly increased risk of recurrence. The pooled RR was 2.46 (95% CI: 2.29–2.64, *p* < 0.001), showing a twofold increase in the risk of recurrence for patients with more advanced disease. This association was consistent across studies, and the heterogeneity was low (*I*^2^ = 0%). This result underscores the importance of staging in predicting recurrence and highlights the need for close follow-up and tailored treatment for patients with advanced stages of the disease (Figure 4).

#### 3.2.4 Association between LVSI positivity and recurrence

**Figure 5:**
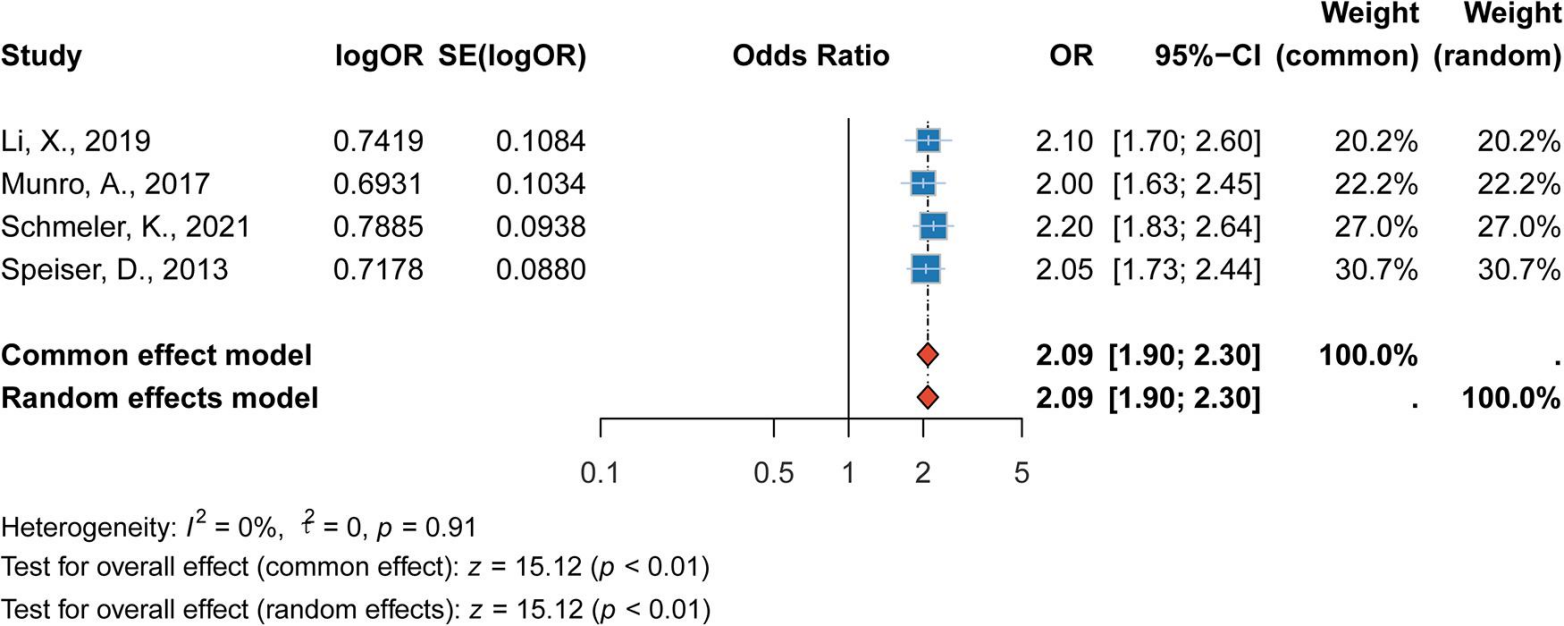
Forest plot of the association between LVSI positivity and recurrence risk.

The presence of lymphovascular space invasion (LVSI) was strongly associated with an increased risk of recurrence. The pooled RR was 2.09 (95% CI: 1.90–2.30, *p* < 0.001), indicating that LVSI-positive patients had more than twice the risk of recurrence compared to LVSI-negative patients. This association was consistent across studies, with minimal heterogeneity (*I*^2^ = 0%). The strong association between LVSI positivity and recurrence risk highlights the importance of LVSI as a prognostic marker in cervical cancer management (Figure 5).

#### 3.2.5 Association between lymph node metastasis and recurrence

**Figure 6:**
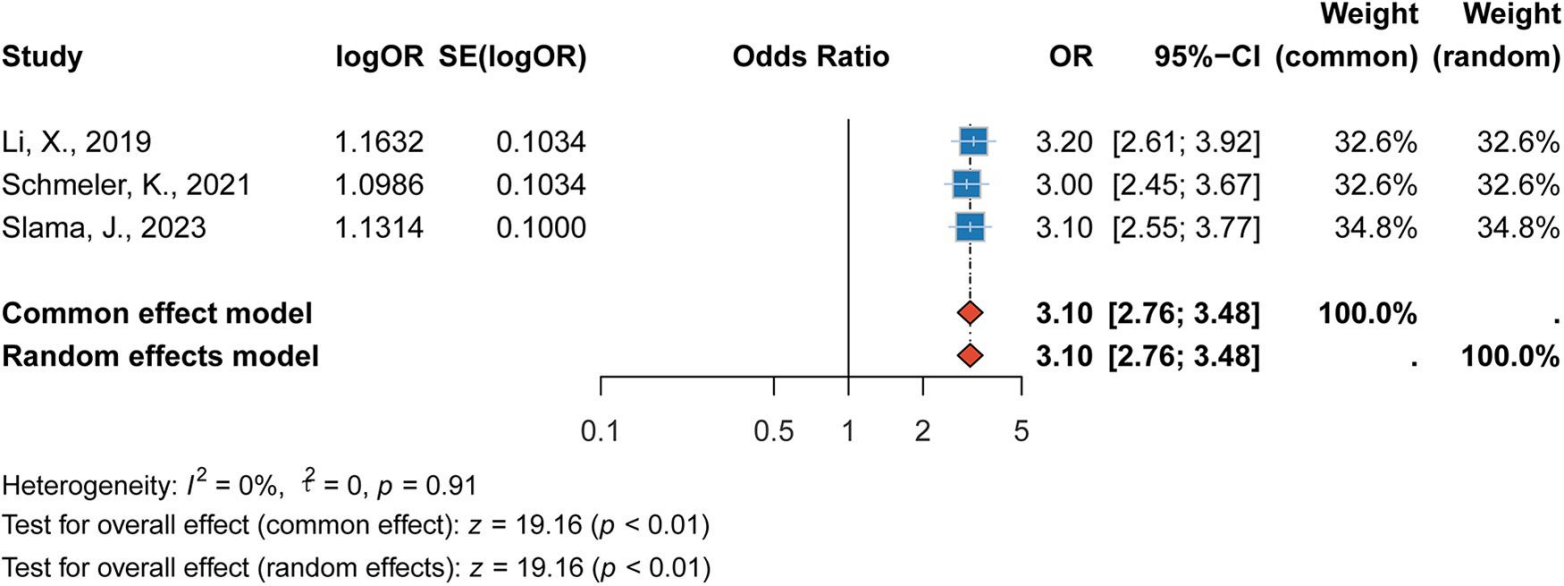
Forest plot of the association between lymph node metastasis and recurrence risk.

Lymph node metastasis was identified as a major risk factor for recurrence in cervical cancer patients. The pooled RR was 3.10 (95% CI: 2.76–3.48, *p* < 0.001), indicating that patients with lymph node metastasis had more than three times the risk of recurrence compared to those without lymph node involvement. The heterogeneity for this risk factor was low (*I*^2^ = 0%). This finding further reinforces the critical role of lymph node metastasis in the prognosis of cervical cancer and the need for comprehensive lymph node evaluation during treatment (Figure 6).

### 3.3 Risk of bias results

Publication bias was evaluated using Begg's test, Egger's test, and funnel plots. Both statistical tests indicated no significant evidence of publication bias across all risk factors analyzed (Table 2). These results suggest that the findings of this meta-analysis are robust and unlikely to be influenced by selective reporting.

**Table 2:** Risk of bias results from begg and egger tests.

Risk factor	Begg test	Egger test
Age ≤30 years	1.00	0.34
Tumor size ≥2 cm	0.29	0.89
Stage > IA	0.57	0.66
LVSI Positive	1.00	0.85
Lymph node metastasis	0.60	0.99

## 4 Discussion

This meta-analysis aimed to identify the key risk factors associated with recurrence in cervical cancer patients who underwent fertility-sparing treatments. Our results highlight the significant impact of age, tumor size, advanced stage, LVSI, and lymph node metastasis on the risk of recurrence. These findings are crucial for clinicians in providing personalized care and follow-up strategies for young women with cervical cancer, balancing the preservation of fertility with the need for effective oncological control.

### 4.1 Age as a risk factor for recurrence

Our analysis found that younger patients, specifically those aged ≤30 years, have more than double the risk of recurrence compared to those aged >30 years (RR: 2.03, 95% CI: 1.89–2.19). This finding aligns with previous studies that suggested age as an independent prognostic factor for recurrence in fertility-sparing treatments for cervical cancer (7, 18). Younger patients may present with more biologically aggressive tumors, which could explain the higher recurrence rates (20, 21). Additionally, the emotional and psychological impact of recurrence in this population is significant, as these women are often at a stage of life where fertility preservation is a priority (22). The underlying biological mechanisms that contribute to the higher recurrence risk in younger patients warrant further investigation, including the role of genetic factors, tumor biology, and immune responses.

### 4.2 Tumor size and recurrence risk

We also found a significant association between tumor size (≥2 cm) and increased recurrence risk (RR: 1.94, 95% CI: 1.82–2.06). Larger tumors are more likely to invade surrounding tissues, and the risk of positive margins after fertility-sparing surgery increases with tumor size (23). This observation has been consistently reported in the literature, where tumor size is recognized as one of the most important prognostic factors in cervical cancer. In clinical practice, international guidelines generally recommend fertility-sparing surgery only for tumors ≤2 cm, reflecting the higher oncologic risks in larger lesions. For patients with tumors >2 cm, fertility preservation should therefore be approached with great caution, and is usually considered inappropriate outside clinical trials or highly selected cases after multidisciplinary evaluation (24).

### 4.3 Advanced stage and recurrence

The results of our meta-analysis underscore the significant risk associated with higher substage within early-stage cervical cancer eligible for fertility-sparing surgery. Patients with stage IA2 or IB1 ≤2 cm compared with IA1 had a pooled RR of 2.46 (95% CI: 2.29–2.64), indicating more than a twofold increase in the risk of recurrence compared to early-stage patients. This finding is consistent with existing literature, which has repeatedly demonstrated that more advanced disease stages are associated with worse prognosis (13, 17). It should be emphasized that fertility-sparing surgery is not appropriate for patients with suspected parametrial involvement, as such cases fall outside accepted indications. Advanced-stage tumors often involve deeper cervical structures and more extensive lymphatic spread, which makes complete surgical resection more challenging and increases the likelihood of residual disease. As such, patients with advanced-stage disease may require more intensive treatment regimens, including adjuvant therapies or comprehensive lymph node evaluation, to reduce the risk of recurrence.

### 4.4 LVSI and recurrence

Our analysis demonstrated a strong association between LVSI positivity and an increased risk of recurrence (RR: 2.09, 95% CI: 1.90–2.30). LVSI is a histopathological feature that indicates the spread of tumor cells into the lymphatic and vascular spaces, suggesting a higher likelihood of metastatic spread (25). This finding is consistent with previous studies that have shown LVSI to be a critical prognostic marker for recurrence in cervical cancer (11). Patients with LVSI positivity require careful monitoring and may benefit from more aggressive treatment strategies, such as adjuvant chemotherapy or radiation therapy, to reduce the risk of recurrence.

### 4.5 Lymph node metastasis and recurrence

Lymph node metastasis was identified as one of the most critical risk factors for recurrence, with a pooled RR of 3.10 (95% CI: 2.76–3.48). This result aligns with the understanding that the presence of lymph node metastasis is a key determinant of prognosis in cervical cancer. It should be emphasized, however, that negative nodal status is a prerequisite for fertility-sparing surgery, and patients with confirmed nodal metastasis are not eligible for this approach. Lymph node involvement significantly worsens the survival outlook, as it indicates the spread of cancer beyond the primary tumor site (26, 27). The observed association in our meta-analysis mainly reflects the prognostic weight of occult nodal disease that may be missed during initial staging or incidentally discovered later. If nodal metastasis is identified, fertility-sparing treatment should be abandoned in favor of standard radical management (16). Early detection of lymph node metastasis is critical, and modern imaging techniques or sentinel lymph node biopsy should be integrated into routine clinical practice for better risk stratification.

### 4.6 Limitations of the study and supplementary analysis of publication bias

Although no significant publication bias was identified in this study through Begg's test, Egger's test, and funnel plot assessment (Table 2), the potential limitations should still be viewed objectively. First, among the 10 included studies, 8 were retrospective cohort studies, and only 2 were prospective observational studies. Retrospective designs are susceptible to “selection bias”; for example, some studies may have prioritized including patients with complete follow-up data, which could lead to results skewed toward “low recurrence risk.” In contrast, the prospective studies had smaller sample sizes, which may limit the generalizability of the results. Second, there were slight differences in the definition of “recurrence” across different studies: 6 studies confirmed recurrence based on “clinical symptoms + imaging (MRI/CT)”; 3 studies additionally included “elevated tumor markers (SCC-Ag)” as an early warning indicator; and 1 study did not clearly specify the diagnostic criteria. Although this study supplemented the details of recurrence diagnosis for 7 studies by contacting the corresponding authors, it was still unable to completely eliminate the minor impact of definitional differences on the pooled analysis. Furthermore, some studies had a relatively short follow-up period (the shortest being 24 months, which may have missed the peak of long-term recurrence of cervical cancer, i.e., “3–5 years after surgery,” and thus had a certain impact on the accuracy of the assessment of long-term recurrence risk.

While this meta-analysis provides important insights into the risk factors for recurrence in fertility-sparing cervical cancer treatment, it is not without limitations. First, the included studies varied in terms of their treatment protocols, follow-up durations, and patient populations. Although we performed a subgroup analysis to assess potential sources of heterogeneity, differences in study design and clinical practices might have influenced the results. Additionally, most studies did not provide complete data on the exact treatment approaches used, such as specific surgical techniques or adjuvant therapies, which could further impact recurrence rates. Another limitation is the potential for publication bias, though our analysis of Begg's and Egger's tests suggested minimal bias in the studies included.

### 4.7 Clinical translation value: recommendations for optimizing existing management guidelines

Despite the aforementioned limitations, the five recurrence risk factors identified in this study (age ≤ 30 years, tumor size ≥ 2 cm, stage > IA, positive LVSI, lymph node metastasis) still have clear clinical translation significance. They can provide evidence-based basis for the update of guidelines in the field of fertility-sparing treatment for cervical cancer. The specific practical recommendations are as follows: ① optimization of preoperative risk stratification: It is recommended to formally include “age ≤ 30 years” in the existing risk stratification system for fertility-sparing treatment. For such patients, the recurrence risk (RR = 2.03) should be clearly informed in writing before surgery, and an enhanced follow-up plan should be formulated—follow-up once every 3 months within 2 years after surgery (compared to once every 6 months for regular patients). In addition to routine gynecological examinations, the follow-up items should include additional pelvic MRI (to assess local lesions) and serum SCC-Ag testing (to monitor tumor load) to achieve early warning of recurrence. ② Refined Management of Tumor Size Thresholds: Further strengthen the evidence-based basis for using “tumor ≤ 2cm” as the core indicator for fertility-sparing treatment (RR = 1.94). Meanwhile, establish a multidisciplinary team (MDT) consultation mechanism for “borderline patients” with tumor sizes ranging from 1.5 to 2.0 cm. The MDT team needs to comprehensively assess the feasibility of surgery by combining pelvic MRI evaluations of tumor invasion depth, whether the internal cervical os is involved, and the LVSI status confirmed by pathological examination. This avoids using tumor size alone as the sole decision-making criterion and reduces the risks of “over-treatment” or “under-treatment”. ③ Clarification of Contraindications and Strengthening of Preoperative Evaluation: It is recommended to explicitly list “LVSI positivity” (RR = 2.09) and “lymph node metastasis” (RR = 3.10) as absolute contraindications for fertility-sparing treatment. During the preoperative evaluation phase, pelvic MRI (to check for enlarged lymph nodes) and sentinel lymph node biopsy (SLNB) should be routinely performed. For SLNB, the “dye + radionuclide dual tracing” technique combined with intraoperative frozen pathological examination is required to minimize the missed diagnosis rate of occult lymph node metastasis; if the biopsy indicates lymph node metastasis, the fertility-sparing plan should be terminated immediately and converted to radical hysterectomy combined with pelvic lymph node dissection. ④ Construction and Application of a Simple Risk Scoring Model: Based on the results of this study, a clinically practical risk scoring model can be constructed—each of the above-mentioned risk factors present scores 1 point, with a total score ranging from 0 to 5 points, among which a score of ≥2 points indicates a high-risk population. For high-risk patients, radical treatment should be prioritized in clinical practice; if patients refuse radical surgery due to strong fertility needs, after filing with the ethics committee, an intensive treatment regimen of “radical trachelectomy + postoperative adjuvant chemotherapy (paclitaxel + cisplatin regimen, once every 3 weeks, for a total of 4 cycles)” should be adopted, and the follow-up period should be extended to 5 years after surgery.The above recommendations are highly consistent with the updated direction of “precision stratification and individualized decision-making” in the recently published cervical cancer management guidelines in the journal Cancers (28). They can provide clinicians with specific operational pathways in balancing “tumor radical cure” and “fertility preservation,” ultimately improving the quality of life and reproductive outcomes of young cervical cancer patients.

## 5 Conclusion

This meta-analysis demonstrates that age ≤30 years, tumor size ≥2 cm, higher substage within early-stage disease, LVSI positivity, and lymph-node metastasis are each associated with increased recurrence after fertility-sparing treatment for cervical cancer. These data reinforce guideline-based selection: fertility-sparing surgery should generally be restricted to tumors ≤2 cm with negative nodes, while suspected parametrial involvement or nodal positivity preclude fertility preservation. Incorporating these variables into preoperative counseling, operative planning, and risk-stratified surveillance may enhance oncologic safety while preserving reproductive potential in appropriately selected patients. Prospective studies with standardized staging, sentinel-node ultrastaging, and molecular profiling are needed to validate risk models and test strategies that reduce recurrence without compromising fertility.

## Data Availability

The raw data supporting the conclusions of this article will be made available by the authors, without undue reservation.
